# Identifying New Risk Associations Between Chronic Physical Illness and Mental Health Disorders in China: Machine Learning Approach to a Retrospective Population Analysis

**DOI:** 10.2196/72599

**Published:** 2025-06-30

**Authors:** Lizhong Liang, Tianci Liu, William Ollier, Yonghong Peng, Yao Lu, Chao Che

**Affiliations:** 1 School of Computer Science and Engineering, Sun Yat-sen University, Guangzhou, China; 2 Affiliated Hospital of Guangdong Medical College Hospital, Zhanjiang, China; 3 Key Laboratory of Advanced Design and Intelligent Computing, Dalian University, 10 Xuefu Street, Dalian, 116622, China, 86 041187402046; 4 Faculty of Science and Engineering, Manchester Metropolitan University, Manchester, United Kingdom; 5 Faculty of Science and Engineering, Anglia Ruskin University, Cambridge, United Kingdom

**Keywords:** chronic physical illnesses, mental health disorders, machine learning, temporal dependence, disease trajectory, comorbidity risk

## Abstract

**Background:**

The mechanisms underlying the mutual relationships between chronic physical illnesses and mental health disorders, which potentially explain their association, remain unclear. Furthermore, how patterns of this comorbidity evolve over time are significantly underinvestigated.

**Objective:**

The main aim of this study was to use machine learning models to model and analyze the complex interplay between mental health disorders and chronic physical illnesses. Another aim was to investigate the evolving longitudinal trajectories of patients’ “health journeys.” Moreover, the study intended to clarify the variability of comorbidity patterns within the patient population by considering the effects of age and gender in different patient subgroups.

**Methods:**

Four machine learning models were used to conduct the analysis of the relationship between mental health disorders and chronic physical illnesses.

**Results:**

Through systematic research and in-depth analysis, we found that 5 categories of chronic physical illnesses exhibit a higher risk of comorbidity with mental health disorders. Further analysis of comorbidity intensity revealed correlations between specific disease combinations, with the strongest association observed between prostate diseases and organic mental disorders (relative risk=2.055, *Φ*=0.212). Additionally, by examining patient subgroups stratified by age and gender, we clarified the variability of comorbidity patterns within the population. These findings highlight the complexity of disease interactions and emphasize the need for targeted monitoring and comprehensive management strategies in clinical practice.

**Conclusions:**

Machine learning models can effectively be used to study the comorbidity between mental health disorders and chronic physical illnesses. The identified high-risk chronic physical illness categories for comorbidity, the correlations between disease combinations, and the variability of comorbidity patterns according to age and gender provide valuable insights into the complex relationship between these two types of disorders.

## Introduction

Contemporary medicine is increasingly focused on understanding the underlying relationships between physical and mental health [1,2]. A growing body of evidence suggests that potential increasing links exist between mental health disorders and chronic physical illnesses. Furthermore, individuals with certain chronic physical illnesses often face increasing challenges relating to their mental health issues, and vice versa [3-5]. Chronic physical illnesses, such as diabetes, cardiovascular disease, and prostate-related diseases, are growing global public health issues. These conditions not only impact the physical health of the patient but also pose a significant challenge at a socio–health care level [6,7]. Mental health disorders such as depression, anxiety, and schizophrenia can significantly affect an individual’s physical health and overall quality of life [8]. The coexistence of chronic physical illnesses and mental health disorders often leads to further health complications. Increasing comorbidity complicates both diagnosis and clinical treatment. It also increases polypharmacy, leading to a reduction in patient life expectancy. Studies have shown that patients with severe mental health disorders as well as type 2 diabetes have a 3‐4 times higher risk of a premature death than that seen in the general population. Cardiovascular disease is one of the leading causes of premature death in this group [9].

Focusing on the physical condition of a person with a mental health disorder prior to its diagnosis is critical as this can provide critical insight and information required to guide preventive and therapeutic interventions. For example, a physical condition that precedes the onset of a particular mental health disorder may share underlying symptoms or pathoetiological mechanisms [10]. Knowing which physical conditions are more prevalent in people diagnosed with a mental health disorder can better inform the introduction of an effective screening tool for “at-risk” populations. It may also help deliver an appropriate intervention to prevent further progression of increased multimorbidity. Such an approach should lead to significant health-associated cost savings and better health outcomes [11].

The complex etiologies and pathophysiological mechanisms that underpin and drive the development of comorbidity between chronic physical illness and mental health disorders have not, as yet, been significantly established or elucidated. A number of recent research studies have started to explore some potential underlying mechanisms of comorbidity. These have been largely through clinical observations or suggested from molecular network analyis [12-16]. Other studies have applied machine learning (ML) models trained through the analysis of large clinical datasets, such as electronic health records. These have now started to identify patterns of comorbidity associated with the development of certain disorders. These have also been applied to predict the likelihood of developing specific comorbidities based on a patient’s past medical history and other relevant characteristics [17-20]. ML methods to predict the progression of disease comorbidities have great potential to ultimately improve accurate medicine and lead to the delivery of appropriate care [21].

To date, most of the studies have been restricted to high-income countries (eg, European countries, the United States, and Australia). Although China represents one of the largest populations in the world, little research has been conducted that relates to the development of comorbidity in people living with diabetes [22]. Given that approximately one-third of the world’s adult population has 2 or more long-term conditions, further research into the relationship between chronic physical conditions and the onset of mental health disorders is now of major importance. It is also important to investigate a wide range of discrete populations in different countries as sociological, cultural, and genetic variations will undoubtedly exist and this heterogeneity can provide valuable insights into the relationships of physical and mental health conditions.

In this study, we investigate the temporal nature of patients’ disease trajectories compared with previous reports that have analyzed comorbidity patterns directly from large-scale data using network analysis [13-15], association rules [23,24], or comorbidity indices [25]. We have focused on exploring the risk relationship that potentially exists between chronic physical illness diagnoses and mental health disorders. This analysis has only been possible through access to the patient’s diagnostic record prior to the diagnosis of a mental health disorder. This permitted insight into each patient’s disease trajectory and the possibility of identifying key factors and patterns that may influence the subsequent development of a mental health disorder.

The primary objective of this retrospective analysis of a large population living in Guangdong, China, was to identify chronic physical illnesses with a higher risk of co-occurrence with mental health disorders. In addition, this study aimed to evaluate and analyze comorbidity patterns between chronic physical illnesses and mental health disorders, as well as assess age and gender differences within such patterns.

## Methods

### Data Analyzed

All patients with a diagnosis of a mental health disorder in the southern region of Guangdong, China, during the study period (January 1, 2016 to December 31, 2022) were included in the analysis. The electronic health record included unique identity, age, gender, hospital admission and discharge dates, and 16 diagnostic codes (including the primary diagnostic code) for each patient. Diagnostic codes were recorded using the *International Classification of Diseases, Tenth Revision* (ICD-10). As more than 20,000 unique and active diagnostic codes now exist in this coding format, it was impractical to analyze each ICD diagnostic code. We therefore processed the codes as 4-digit codes and filtered these into two categories: (1) chronic physical illnesses and (2) mental health disorders. Based on existing published literature and studies, we conducted prevalence analysis for the 60 chronic diseases proposed by Vetrano et al [26]. Chronic physical illnesses with a prevalence rate greater than or equal to 1% (ICD-10 codes for diseases not beginning with F) were selected for inclusion into the study [16]. Analysis of prevalence was also performed for mental health disorders [8] to decide which conditions should be included in the study. Table 1 illustrates the age-sex groupings and Table 2 shows the prevalence and number of mental health disorders.

**Table 1.** Age-sex groups (N=46,649).

Age group (years)	Total participants (N=46,649), n (%)	Female (n=22,313), n (%)	Male (n=24,336), n (%)
0‐18	2835 (6.08)	868 (3.98)	1967 (8.08)
18‐45	12,732 (27.29)	4836 (21.67)	7896 (32.45)
45‐60	9632 (20.64)	5270 (23.62)	4362 (17.92)
60‐80	13,764 (29.51)	7483 (33.54)	6281 (25.81)
>80	7686 (16.48)	3856 (17.28)	3830 (15.74)

**Table 2.** The prevalence and number of mental health disorders (N=46,649).

Diagnosis of major mental health disorders	Disease name	Prevalence, n (%)
F00-F09	Organic mental disorders	12,273 (26.31)
F10-F19	Mental and behavioral disorders due to psychoactive substance use	1094 (2.35)
F20, F22, F24, F25, F28	Schizophrenia and delusional diseases	14,634 (31.37)
F30-F34, F38, F39	Depression and mood diseases	3601 (7.72)
F40-F45, F48	Neurotic stress–related and somatoform diseases	15,877 (34.04)
F50	Eating disorders	82 (0.18)
F51.0-F51.3	Sleep disorders_F	612 (1.31)
F60	Specific personality disorders	29 (0.06)
F70-F79	Mental retardation	3431 (7.35)
F84	Pervasive developmental disorders	622 (1.33)
F90-F98	Behavioral and emotional disorders with onset usually occurring in childhood and adolescence	229 (0.49)

A total of 41 chronic physical illnesses were included: cerebrovascular disease, esophagus stomach and duodenum diseases, hypertension, dorsopathies, other metabolic diseases, dyslipidemia, ischemic heart disease, heart failure, other cardiovascular diseases, other respiratory diseases, anemia, chronic obstructive pulmonary disease, emphysema chronic bronchitis, diabetes, dementia, other genitourinary diseases, prostate diseases, other musculoskeletal and joint diseases, other neurological diseases; ear, nose, and throat diseases; cardiac valve diseases, osteoporosis; chronic pancreas, biliary tract, and gall bladder disease; sleep disorders, solid neoplasms, osteoarthritis and other degenerative joint diseases, inflammatory arthropathies, epilepsy, cataract and other lens diseases, colitis and related diseases, thyroid diseases, chronic liver diseases, atrial fibrillation, chronic ulcer of the skin, chronic infectious diseases, Parkinson and parkinsonism, migraine and facial pain syndromes, blood and blood-forming organ diseases, chronic kidney diseases, allergy, peripheral neuropathy, and other eye diseases.

In addition, 8 mental health disorders were included: neurotic stress-related and somatoform diseases, schizophrenia and delusional diseases, organic mental disorders, depression and mood diseases, mental retardation, mental and behavioral disorders due to psychoactive substance use, pervasive developmental disorders, and sleep disorders (ICD code starts with F).

### Data Preprocessing

#### Overview

Mental health disorders were taken as the target disease. Data were extracted from the hospital admission and discharge records of each patient. The disease trajectory over chronological time was then ordered and used as the predictor variable for that particular disease. All data analyses and visualizations were conducted in Python (version 3.8). The main steps of data preprocessing are described in the next section.

#### Missing Value Handling

After including only chronic physical diseases with a prevalence of ≥1% (based on the criteria by Vetrano et al, 2020 [26]) and excluding rare diseases to reduce noise, we further addressed missing fields in the diagnostic records (such as age and gender) by using the deletion method (retaining only complete data). Since the data in this study are derived from patient admission records, and the missing value ratio is below 5%, this approach was deemed appropriate.

#### Data Cleaning

This step consisted of the filtering and screening of 348,563 admission and discharge records of 102,409 patients with mental health disorders from 2016 to 2022. We specifically selected patients who had at least 2 admission and discharge records. Subsequently, ICD-10 coding standardization was used to screen for valid diagnoses, exclude duplicate records (such as the same diagnosis across multiple hospitalizations), and perform ICD coding. This resulted in a “cleaned” dataset of 268,588 admission and discharge records from 46,649 patients, achieving a screening rate of 77.05%.

#### Dataset Construction

The “cleaned” patient admission records were then converted into a dataset using classification and labeling. The specific steps are as follows:

1. Time dependency of admission records: We extracted the hospital records of each patient in chronological order. The first record without the target disease was used as a feature, and the subsequent record was converted into a classification label (“1” for the presence of the target disease, “0” for other diseases), until the target disease was identified. This method was then applied to process the hospital records of all other patients in the study.
2. Exclusion of rare data: Records with fewer than 5 occurrences were removed to further process the dataset, preventing excessively sparse features and avoiding model overfitting. After this step, the dataset contained more than 23,000 records. Then, 70% of the dataset was used as the training set, and the remaining 30% was used as the test set for performance comparison.

### ML Classification Models Used for Assessing Risk Diseases

Four ML algorithms were used to model patient disease trajectories based on temporal dependencies, study the risk of comorbidity between chronic physical illnesses and mental health disorders based on patient disease trajectories, and assess chronic physical illnesses that increase the risk of mental health disorder development.

**Figure 1.**
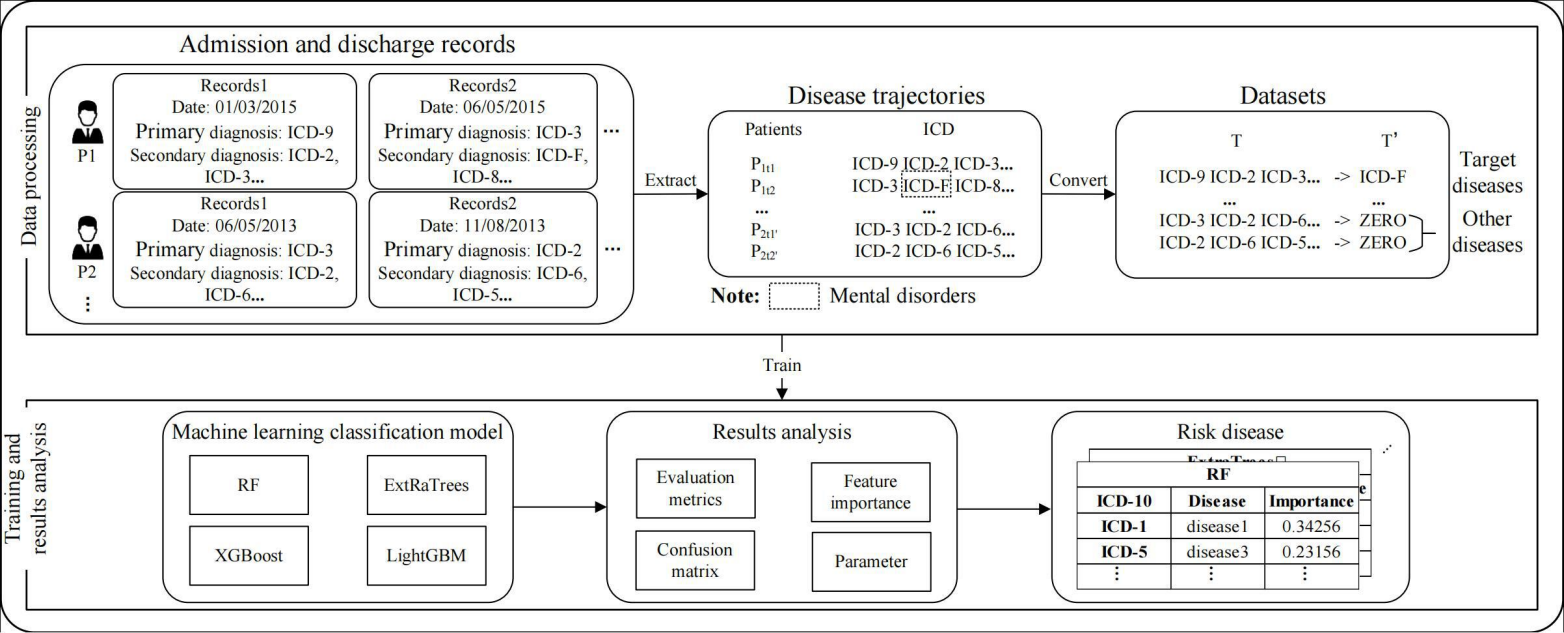
Framework for assessing risk diseases using patient trajectories and classification machine learning models. ExtRaTrees: extremely randomized trees; ICD: International Classification of Diseases; light GBM: light gradient boosting machine; RF: random forest; XGBoost: extreme gradient boosting.

These ML models were (1) random forest (RF), (2) extremely randomized trees (ExtRaTrees), (3) light gradient boosting machine (light GBM), and (4) extreme gradient boosting (XGBoost). The main framework for the analysis framework is described in Figure 1.

By using ML classification models, we modeled patient disease trajectories with a focus on temporal dependencies. This allowed a thorough exploration of the risk relationships between chronic physical illnesses and mental health disorders before patients were subsequently diagnosed with mental health conditions. This uncovered potential risk conditions that preceded their diagnosis. Traditional risk models often overlook the study of patient comorbidities in the context of temporal dependencies. The approach we used provided a better understanding of such relationships between different diseases over a patient’s life course, particularly in the period prior to the diagnosis of mental health conditions and disorders.

Given the low frequencies of some conditions identified in the dataset, we focused on developing models that can deal with sparse data for classification in anticipation of a more significant performance advantage. Although deep learning models have shown outstanding performance in certain scenarios, we prioritized model interpretability and robustness against sparse data. Additionally, to assess the risk factors contributing to mental disorders, the top 10 most important features (ICD codes) from each model’s classification process were evaluated, which is why comparative models were not included in this analysis. The profiles of the models used are summarized in Table 3.

In our study, we optimized the parameters of the ML model using 5-fold cross-validation. Additionally, oversampling and hyperparameter tuning were used to address the issue of class imbalance. The model was then trained using the optimal parameters. Then, the 10 most important features (ICD codes) of the model classification process were evaluated and categorized to obtain risk diseases, and these were subsequently analyzed for comorbid combinations with mental health disorders.

**Table 3.** Description of the model and similar literature.

Model	Model description	Advantage	Similar documents
Random forest	An integrated learning algorithm based on multiple decision trees. The final output is determined by voting or averaging the outputs of these decision trees.	Resistance to overfitting; large data handling; feature importance assessment; easy parallelization; missing value handling.	[14,20,21,27]
Extremely randomized trees	Integrated learning algorithms based on decision trees are similar to random forest analysis, but differ by choosing the cut points randomly within a random range when constructing the decision tree.	Superior resistance to overfitting; high computational efficiency; robustness to outliers and noise; feature importance assessment.	[27,28]
Light gradient boosting machine	Gradient boosting tree-based machine learning algorithms achieve efficient processing of large data by using a histogram-based decision tree algorithm and parallelized training. This approach supports the processing of high-dimensional sparse features and class features with a fast training speed, low memory consumption, and superior accuracy.	Efficient training speed; low memory footprint; high accuracy; support for parallelized learning; missing value handling; flexible parameter tuning.	[29,30]
Extreme gradient boosting	This approach is similar to light gradient boosting machine, but excels in handling large-scale data and complex features, using unique techniques such as regularization, parallelized processing, and custom loss functions to improve model accuracy and generalization.	High performance; regularization; missing value handling; flexibility; feature importance assessment; interpretability; rich hyperparameters.	[20,21,30]

### Data Analysis Methods

For the risk of diseases assessed in the classification model, we used the relative risk (RR) and the *Φ* correlation coefficient (phi correlation coefficient) to measure the intensity of comorbidity between a physical condition and a particular type of mental health disorder [12,15]. Since we were more interested in closely related disease combinations, mutually exclusive disease combinations with negative comorbidity intensity or no comorbidity intensity (RR≤1 or *Φ*≤0) were excluded [16]. The formulae for RR and *Φ* correlation coefficient are shown here:


$$RR_{ij}=\frac{C_{ij}\;N}{P_{i}\;P_{j}}$$

$$\Phi_{ij}=\frac{C_{ij}N-P_{i}P_{j}}{\sqrt{P_{i}P_{j}(N-P_{i})(N-P_{j})}}$$

Where *C_ij_* is the number of patients affected by the 2 diseases, *N* is the total number of patients in the study population, and *P_i_* and *P_j_* are the number of patients with disease *i* and *j*, respectively.

In addition, as there may be potential effects of age and sex on patient comorbidity [16,31-33] and also to obtain more consistent and reliable estimates, we grouped patients by age in year intervals (0‐18, 18-45, 45‐60, 60-80, >80 years) and sex; these were used for further analyses. Mental health disorders were broadly classified into 11 categories; prevalence statistics were performed after classification. Eight mental disorders with prevalence rates greater than or equal to 1% were included in the scope of this analysis.

### Ethical Considerations

Ethics approval for this study was obtained from the Clinical Research Ethics Committee of Affiliated Hospital of Guangdong Medical University (number KT2023-138-01). Written and oral informed consent was obtained from the participants. Additionally, for minors, informed consent was also obtained from their parents or legal guardians. This study used data that were anonymized and no financial compensation was provided to participants.

## Results

### The ML Classification Models for Assessing Risk Diseases

To explore the risk associated with analyzing the comorbidity of chronic physical illnesses and mental health disorders based on patient disease trajectories, we randomly sampled from the dataset to create a baseline dataset. Subsequently, we used 4 ML methods for modeling, with detailed parameters as follows:

- RF: criterion=gini, max feature=sqrt, number of estimators=500, class_weight=balanced
- EXtraTrees: number of estimators=500, class_weight=balanced
- LightGBM: Objective=binary, is_unbalance=True, metric=f1, max_depth=5, num_leaves=31, learning_rate=0.01, reg_alpha=0.9, reg_lambda=1, num_iterations=5000
- XGBoost: objective=binary:logistic, max_depth=6, learning_rate=0.01, n_estimators=5000, colsample_bytree=0.4, subsample=0.8

To evaluate the performance of the model, we used accuracy, precision, recall, *F*_1_-score, and area under the curve (AUC) as evaluation metrics. Accuracy is a basic metric used to evaluate the performance of classification models. This evaluates the ratio of the number of samples correctly predicted by the model relative to the total number of samples. Precision was evaluated for the prediction of results and indicates the proportion of truly positive samples among the samples with positive predictions. Recall was also ascertained for the original sample and indicated how many of the positive classes in the sample were correctly predicted. Since recall and precision are relative concepts, each with its own focus on the identification process of positive samples, the *F*_1_-score was used to combine precision and recall and provide a comprehensive assessment of the accuracy and completeness of the model. A confusion matrix is a 2D table that provides an intuitive way to understand the performance of the model and measure the performance of the classification model. AUC refers to the area under the receiver operating characteristic (ROC) curve, which is a performance metric used to evaluate how well the model distinguishes between the 2 classes (positive and negative). The evaluation metrics formula is shown below:


$$\text{Accuracy}=\frac{TP+TN}{TP+FN+TN+FP}$$

$$\text{Precision}=\frac{TP}{TP+FP}$$

$$\text{Recall}=\frac{TP}{TP+FN}$$

$$F1\text{-}Score=\frac{2\times \text{Precision}\times \text{Recall}}{\text{Precision}+\text{Recall}}$$

Where *TP*: true example, *FN*: false negative example, *FP*: false positive example, *TN*: true negative example.

**Figure 2.**
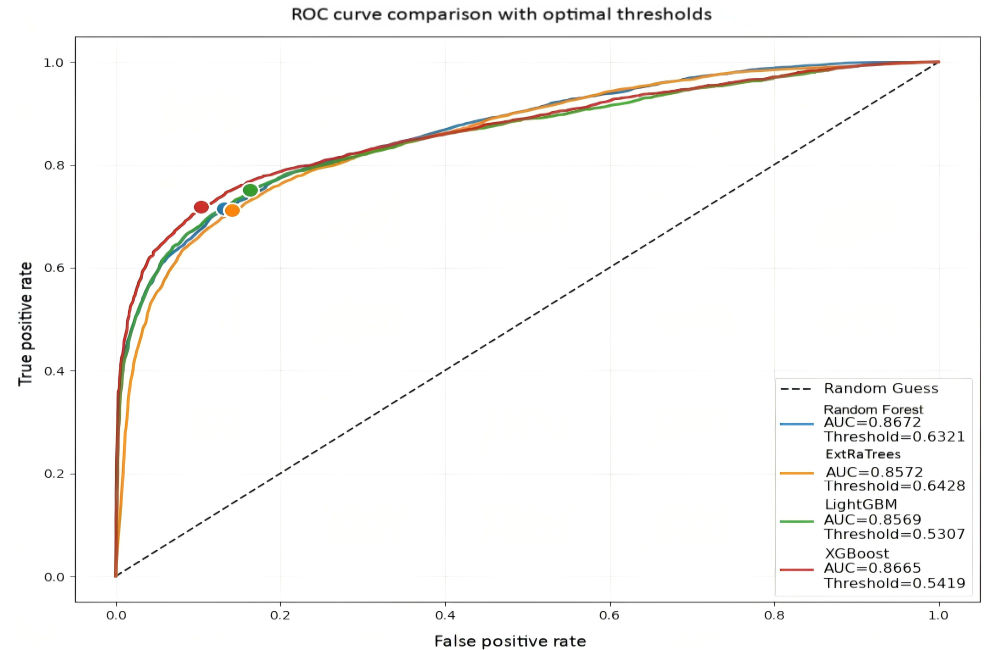
ROC curve comparison with optimal thresholds. AUC: area under the curve; LightGBM: light gradient boosting machine; RF: random forest; ROC: receiver operating characteristic; XGBoost: extreme gradient boosting.

The performance estimates of the 4 classification models are shown in Table 4 and Figure 2, where XGBoost has the highest accuracy (0.8201), RF has the lowest accuracy (0.7597), and RF has the highest recall (0.7750) and AUC (0.8672). Overall, XGBoost has the best *F*_1_ value (0.7815) among the 4 methods. The difference between XGBoost and Light GBM results in this experiment is not large, due to their ability to deal with sparse features. However, XGBoost can be more flexible and accurate when dealing with nonlinear interactions between features by using second-order derivatives to capture the interactions between features.

**Table 4.** Performance of the 4 models for the classification of mental health disorders.

	Accuracy	Precision	Recall	*F*_1_-score	Area under the curve
Random forest	0.7888	0.7597	0.7750	0.7672	0.8672
Extremely randomized trees	0.7855	0.7635	0.7571	0.7603	0.8572
Light gradient boosting machine	0.7978	0.7882	0.7520	0.7697	0.8569
Extreme gradient boosting	0.8125	0.8201	0.7464	0.7815	0.8665

The first 10 important characteristics (ICD codes) of the classification process of each model were evaluated. The categories to which they belonged were queried to obtain the risk diseases affecting mental health disorders (see Table 5). Twelve different ICD codes, belonging to 10 categories of chronic physical illnesses, were obtained by taking into account the results of the evaluation of the 4 models. Eight ICD codes were present in the scores of each model, although the ranking of the scores for these 8 ICD codes varied slightly from model to model. Such commonality suggests that these 8 ICD codes are associated with a higher risk of mental health disorders. The 8 ICD codes fall into 7 categories of chronic physical illnesses: heart failure, hypertension, cerebrovascular disease, ischemic heart disease; esophagus, stomach, and duodenum diseases; prostate disease, and diabetes. Of these 7 categories of chronic physical illnesses, 4 of them (heart failure, ischemic heart disease, hypertension, and cerebrovascular disease) could be broadly classified as cardiovascular diseases and are among the risk factors for mental health disorders. Studies have indicated that mental health disorders, particularly psychological comorbidities such as depression and mood disorders, neurotic stress and somatoform disorders, and substance use disorders have a high prevalence and negative impact among patients with cardiovascular diseases. For instance, the prevalence of depression or neurotic stress–related and somatoform disorders is several times higher in patients with heart failure compared to the general population and may be associated with cognitive issues [4,5]. Individuals with symptoms related to esophagus, stomach, and duodenum diseases, such as pain, dyspepsia, and dietary restrictions, may experience heightened anxiety, depression, and psychological distress. These psychological challenges, in turn, have an impact on their overall psychological well-being. For instance, in conditions like gastroesophageal reflux disease (GERD), the prevalence of psychosocial disorders is higher in GERD patients compared to those without GERD [34,35]. In the case of diabetes, the long-term burden of chronic disease, lifestyle adjustments, medications, and psychosocial pressures can elevate the risk of developing anxiety and depression. Evidence suggests that individuals with diabetes are more susceptible to common psychiatric disorders, particularly mixed anxiety and depression [36,37].

Based on the scoring results of the model with the best categorization performance (XGBoost) as well as those of other models, we identified 3 categories of chronic physical illnesses (anemia, other metabolic disorders, and dyslipidemia) that were scored as risk diseases closely related to mental health disorders. Despite the fact that these 3 categories of chronic physical illnesses ranked at the bottom of the scoring list, they still contributed a very high level of risk. For example, in the case of anemia, this can lead to a decline in physical activity and general fatigue; it can also affect mental status and emotional stability. In iron deficiency, anemia can result in a loss of iron-containing enzymes and proteins during the development of the central nervous system. This can lead to an increased risk of mental health disorders developing. These can include mood disorders, autism spectrum disorders, attention deficit hyperactivity disorder, and developmental disorders [38]. However, the comorbidity and risk between other metabolic disorders and dyslipidemia and mental health disorders is not supported by the literature and requires further research attention to investigate their associated mechanisms.

**Table 5.** Risk diseases for mental disorders in classification models.

Random forest			Extremely randomized trees			Extreme gradient boosting			Light gradient boosting machine		
ICD-10^a^ code	Disease	Weight score (importance)	ICD-10 code	Disease	Weight score (importance)	ICD-10 code	Disease	Weight score (importance)	ICD-10 code	Disease	Weight score (importance)
I50.9	Heart failure	0.048467	I50.9	Heart failure	0.048778	I10.x	Hypertension	0.010559	I50.9	Heart failure	3069
I25.1	Ischemic heart disease	0.044654	I25.1	Ischemic heart disease	0.043862	K29.5	Esophagus, stomach, and duodenum diseases	0.007317	I10.x	Hypertension	2833
I10.x	Hypertension	0.033319	I10.x	Hypertension	0.033509	I25.1	Ischemic heart disease	0.006866	I67.2	Cerebrovascular disease	2470
N40.x	Prostate diseases	0.027458	N40.x	Prostate diseases	0.027809	I50.9	Heart failure	0.006414	I25.1	Ischemic heart disease	2104
K29.5	Esophagus, stomach, and duodenum diseases	0.025265	K29.5	Esophagus, stomach, and duodenum diseases	0.024969	I67.2	Cerebrovascular disease	0.006269	I69.3	Cerebrovascular disease	1984
E11.9	Diabetes	0.020739	E11.9	Diabetes	0.020313	I69.3	Cerebrovascular disease	0.006174	K29.5	Esophagus, stomach, and duodenum diseases	1954
I67.2	Cerebrovascular disease	0.020462	I69.3	Cerebrovascular disease	0.019940	E11.9	Diabetes	0.006115	N40.x	Prostate diseases	1821
I69.3	Cerebrovascular disease	0.020399	I67.2	Cerebrovascular disease	0.019592	N40.x	Prostate diseases	0.005924	E77.8	Other metabolic diseases	1581
E78.5	Dyslipidemia	0.017025	E78.5	Dyslipidemia	0.016730	D64.9	Anemia	0.005724	D64.9	Anemia	1575
I63.9	Cerebrovascular disease	0.016396	I63.9	Cerebrovascular disease	0.016353	E77.8	Other metabolic diseases	0.005624	E11.9	Diabetes	1368

a ICD-10: *International Classification of Diseases, Tenth Revision*.

### Comorbidity Analysis of Risk Diseases and Mental Health Disorders

**Figure 3.**
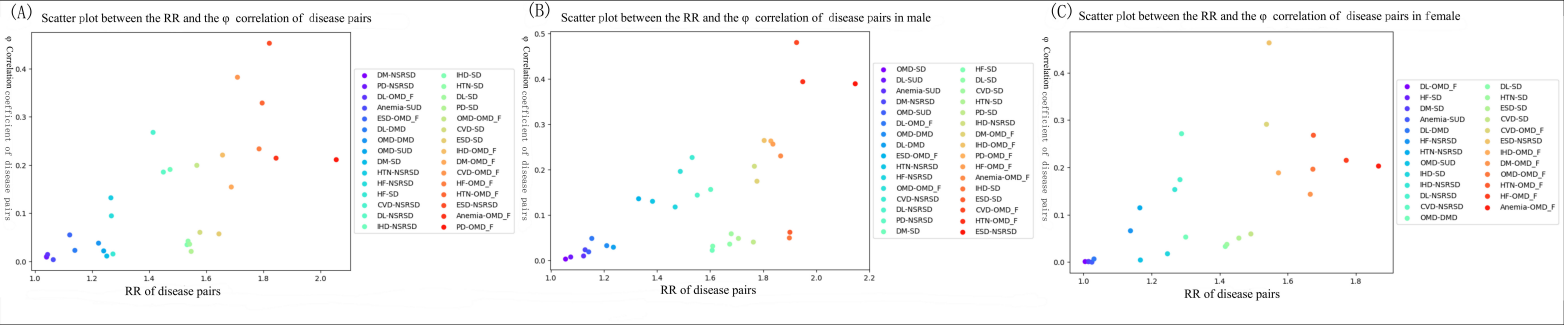
Scatter plot between RR and Φ correlation coefficient for disease combinations (the larger the RR value, the more reddish the scatter color). (**A**) All patient populations, (**B**) male patient populations, and (**C**) female patient populations. CVD: cerebrovascular diseases; DL: dyslipidemia; DM: diabetes; DMD: depression and mood disorders; ESD: esophagus, stomach, and duodenum diseases; HF: heart failure; HTN: hypertension; IHD: ischemic heart disease; MR: mental developmental disorders; NSRSD: neurotic stress–related and somatoform diseases; OMD: other metabolic diseases; OMD_F: organic mental disorders; PD: prostate diseases; PDD: pervasive developmental disorders; RR: relative risk; SD: sleep disorders_F; SDD: schizophrenia and delusional disorders; SUD: mental and behavioral disorders due to psychoactive substance use.

To measure the intensity of comorbidity between risk diseases and a particular class of mental health disorders, we identified 30 different disease combinations with comorbidity intensity (RR>1 and *Φ*>0, as detailed in Figure 3A) from the complete dataset of patients with mental health disorders. The disease combinations with the highest comorbidity intensity were prostate disease and organic mental disorders (including symptoms; RR=2.055, *Φ*=0.212). The disease combinations with the highest comorbidity intensity were prostate disease and organic mental disorders (RR=2.055, *Φ*=0.212). Within the 10 highest risk disease combinations in terms of comorbidity intensity (RR>1.2 and *Φ*>0. 1), 7 combinations included organic mental disorders (hypertension, cerebrovascular disease, anemia, heart failure, prostate disease, ischemic heart disease and diabetes, ischemic heart disease, and other metabolic diseases). A further 3 combinations included neurotic stress–related and somatoform diseases (related to esophagus, stomach, and duodenum diseases and ischemic heart disease). The combinations of the 10 risk diseases with the highest intensity of comorbidity with mental health disorders are shown in Table 6.

**Table 6.** Combinations of 10 risk diseases with the highest intensity of comorbidity with mental health disorders.

Comorbidity combinations	Comorbidity intensity
Prostate diseases and organic mental disorders	RR^a^=2.055, *Φ*=0.212
Anemia and organic mental disorders	RR=1.843, *Φ*=0.214
Esophagus, stomach, and duodenum diseases and neurotic stress–related and somatoform diseases	RR=1.821, *Φ*=0.454
Hypertension and organic mental disorders	RR=1.794, *Φ*=0.329
Heart failure and organic mental disorders	RR=1.785, *Φ*=0.234
Cerebrovascular disease and organic mental disorders	RR=1.708, *Φ*=0.382
Diabetes and organic mental disorders	RR=1.687, *Φ*=0.154
Ischemic heart disease and organic mental disorders	RR=1.656, *Φ*=0.221
Other metabolic diseases and organic mental disorders	RR=1.567, *Φ*=0.199
Dyslipidemia and neurotic stress–related and somatoform diseases	RR=1.448, *Φ*=0.186

a RR: relative risk.

To analyze gender differences in comorbidity patterns, the entire dataset were divided into 2 groups based on gender to identify 32 male and 25 female comorbidity combinations. In the male patient population, the comorbidity combinations with the highest comorbidity intensity were esophagus, stomach, and duodenum diseases and neurotic stress–related and somatoform disorders (RR=2.146, *Φ*=0.391). Within the 10 disease combinations exhibiting the highest comorbidity intensity (RR>1.2 and *Φ*>0.1), 7 combinations included organic mental disorders (ie, related to prostate diseases, anemia, hypertension, heart failure, cerebrovascular diseases, diabetes, ischemic heart disease, and other metabolic diseases), and 3 combinations included neurotic stress–related and somatoform diseases (related to esophagus, stomach, and duodenum diseases, ischemic heart disease, and prostate diseases).

In contrast, the combination of comorbidities with the highest comorbidity intensity in the female patient population was anemia and organic mental disorders (RR=1.867, *Φ*=0.203). Within the 10 highest disease combinations (RR>1.2 and *Φ*>0.1) in terms of comorbidity intensity, 7 combinations included organic mental disorders (associated with anemia, heart failure, hypertension, other metabolic diseases, diabetes, ischemic heart disease, and cerebrovascular diseases) and 3 combinations included neurotic stress–related and somatoform diseases (associated with esophagus, stomach, and duodenum diseases, cerebrovascular diseases, and dyslipidemia). The listed comorbidities all had RR>1. 2 and *Φ*>0.1, as shown in Figure 3B and Figure 3B).

### Gender and Age Differences in Comorbidity Patterns

**Figure 4.**
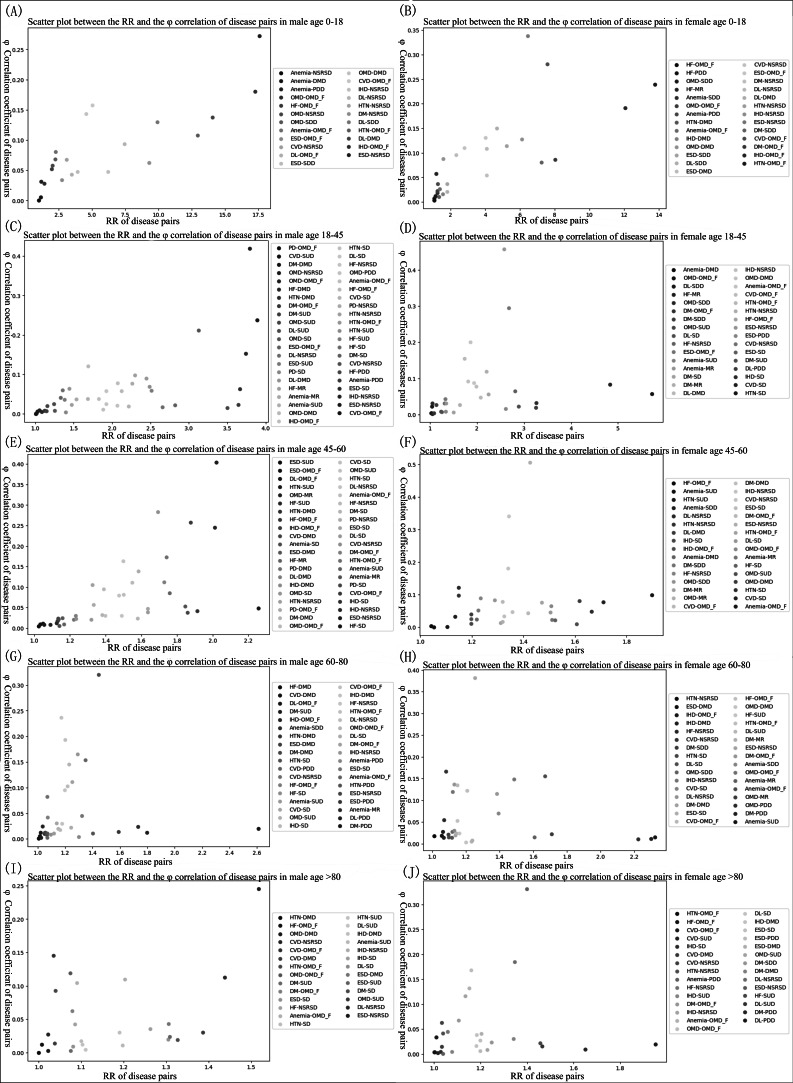
Scatter plot between RR and Φ correlation coefficients for disease combinations within each subgroup. All points in the plot have RR>1 and Φ>0. (**A**) Age 0‐18 years, male sex. (**B**) Age 0‐18 years, female sex. (**C**) Age 18‐45 years, male sex. (**D**) Age 18‐45 years, female sex. (**E**) Age 45‐60 years, male sex. (**F**) Age 45‐60 years, female sex. (**G**) Age 60‐80 years, male sex. (**H**) Age 60‐80 years. CVD: cerebrovascular diseases; DL: dyslipidemia; DM: diabetes; DMD: depression and mood disorders; ESD: esophagus, stomach, and duodenum diseases; HF: heart failure; HTN: hypertension; IHD: ischemic heart disease; MR: mental developmental disorders; NSRSD: neurotic stress–related and somatoform diseases; OMD: other metabolic diseases; OMD_F: organic mental disorders; PD: prostate diseases; PDD: pervasive developmental disorders; RR: relative risk; SD: sleep disorders_F; SDD: schizophrenia and delusional disorders; SUD: mental and behavioral disorders due to psychoactive substance use.

As age and gender were potential factors affecting patients’ health, we divided the complete dataset of patients with mental health disorders into 10 groups according to gender (male and female) and age (0‐18, 18‐45, 45‐60, 60‐80, and >80 years). Significant comorbidity combinations are detailed in Figures 4A-J.

The combinations of comorbidities with comorbidity intensity in all groups were as follows: (1) heart failure and organic mental disorders, (2) ischemic heart disease and neurotic stress–related and somatoform diseases, (3) hypertension and organic mental disorders, (4) esophagus, stomach, and duodenum diseases and neurotic stress–related and somatoform diseases, (5) anemia and organic mental disorders, and (6) other metabolic diseases and organic mental disorders.

Although the previously mentioned 6 comorbidity combinations showed different comorbidity intensities in different age-sex groups of patients, they exhibited high comorbidity intensity in the patient groups, as shown in Table 7.

There are some comorbidity combinations that show a high comorbidity intensity only within certain age-sex groups (as shown in Table 8). In addition, some comorbidity combinations maintained a high comorbidity intensity in either the male or female patient populations (eg, esophagus, stomach, and duodenum diseases and neurological stress–related and somatoform diseases and cerebrovascular diseases and organic mental disorders were found to have a strong comorbidity intensity in all age subgroups of the male patient population, whereas in the age-specific subgroups of the female patient population, only esophagus, stomach, and duodenum diseases and neurotic stress–related and somatoform diseases had a high comorbidity intensity).

**Table 7.** The patient groups in which the 10 comorbidity combinations show a robust comorbidity intensity (relative risk>1.2 and *Φ*>0.1 within the group).

Comorbidity combinations	Patient populations
Esophagus, stomach, and duodenum diseases and neurotic stress–related and somatoform diseases	All age-sex groups
Ischemic heart disease and neurotic stress–related and somatoform diseases	Female patients aged 0‐18 years and male patients aged 18‐45 years
Hypertension and organic mental disorders	Male and female patients aged 0‐18 years
Esophagus stomach and duodenum diseases and neurotic stress–related and somatoform diseases	Male and female patients aged 0‐18 years
Cerebrovascular diseases and organic mental disorders	Male and female patients aged 0‐18 years
Cerebrovascular diseases and neurotic stress–related and somatoform diseases	Male patients aged 18‐45 years and female patients aged 0‐18 years
Anemia and organic mental disorders	Male and female patients aged 60‐80 years
Other metabolic diseases and organic mental disorders	Male and female patients aged 60‐80 years

**Table 8.** Comorbidity combinations with high comorbidity intensity within specific age-sex groups.

Patient populations	Comorbidity combinations	Comorbidity intensity
Male patient population aged 0‐18 years	Dyslipidemia and schizophrenia and delusional diseases	RR^a^=9.897, *Φ*=0.129
Female patient population aged 0‐18 years	Esophagus, stomach, and duodenum diseases and organic mental disorders	RR=4.019, *Φ*=0.131
Male patient population aged 45‐60 years	Prostate diseases and neurotic stress–related and somatoform diseases	RR=1.584, *Φ*=0.139
Male patient population aged 45‐60 years	Heart failure and neurotic stress–related and somatoform diseases	RR=1.547, *Φ*=0.112

a RR: relative risk.

## Discussion

### Principal Results

The potential mechanisms underlying comorbidity are helpful in our understanding of diagnosis and prevention and control in clinical settings. This study analyzes the risk of comorbidity between chronic physical illnesses and mental health disorders. In addition, it importantly examines such comorbidity in the context of both age and gender differences in 46,649 patients with mental health disorders.

In terms of disease prediction methods, patient clinical trajectories were used to analyze the risk of comorbidity between chronic physical illnesses and mental health conditions; ten categories of physical illnesses with a high risk for developing mental health comorbidity were evaluated. A high intensity of comorbidity was found between physical conditions and organic stress–related and somatoform diseases as well as neurotic stress–related and somatoform diseases.

We found that comorbidities affect all age and gender groups, with more combinations of comorbidities, along with significant stronger comorbidity strengths in the 18‐45 and 45‐60 year old male patient populations. In particular, the interaction between chronic physical illnesses and mental disorders was stronger in the group of male patients aged 45‐60 years (most disease combinations with RR>1.2 and *Φ*>0.1).

To our knowledge, this is the first study in the literature to use regional patient longitudinal medical record data rather than self-reported survey data to assess risk diseases for comorbidity between chronic physical illnesses and mental health disorders and to also analyze age and gender differences in comorbidity patterns.

### Comorbidity Between Chronic Physical Illnesses and Mental Health Disorders and Potential Common Mechanisms

We identified chronic physical illnesses with a higher risk of being associated with mental health disorders. Some of these exhibited a stronger correlation as well as showing frequent comorbidities. Additionally, a number of comorbid combinations displayed significant comorbidity intensity between a chronic physical illness and mental health disorder(s), for example, heart failure with organic mental disorders; anemia with organic mental disorders; esophagus, stomach, and duodenum diseases with neurotic stress–related and somatoform diseases; and hypertension with organic mental disorders. These were more frequent than their expected randomized occurrence in high-income countries or regions [39]. For example, the analysis by Cannon et al [5] found that patients with heart failure have a higher relative risk and prevalence of organic mental disorders (Alzheimer disease, etc) and also have concurrent cognitive problems.

Chronic physical illnesses and mental health disorders suggest a risk for physical and mental comorbidity. It may be that chronic physical illnesses are associated with an increased risk of mental health disorders and vice versa. To fully appreciate the sequence of such events and the temporal onset, there is a need to retrospectively analyze a longitudinal cohort of people where clinical data are ethically available to analyze. Ideally, such data should be “real-world data” that fully reflects the demographic and ethnic makeup and structure of the study area population. Furthermore, the high-quality data should be collected over a long duration. This is not always possible in prospective studies where a sampling bias distorts the relevance to the whole population.

In terms of the pathogenesis of chronic physical illness and mental health disorder comorbidity, there are 4 possible causes:

1. Chronic physical illness and mental disorder comorbidity share common biological mechanisms, such as inflammatory and immune pathways or dysregulation of the neuroendocrine system. For example, chronic physical illnesses (eg, diabetes) can activate proinflammatory cytokines (eg, interleukin-6, tumor necrosis factor alpha), affecting blood-brain barrier permeability and leading to neuroinflammation [37].
2. The mechanism of dysregulation of the regulatory systems may mean that patients with chronic pain also experience symptoms of depression and anxiety, which may be related to an imbalance in the neuromodulatory system.
3. Psychosocial factors and lifestyle behaviors (eg, suboptimal diet, lack of exercise, poverty and deprivation) can influence both chronic physical illnesses and mental disorders. For example, chronic stress and anxiety can lead to an increased inflammatory response in the body, increasing the risk of heart disease or diabetes. Individuals who have a history of a suboptimal diet are likely to be deficient in vitamins and critical minerals as well as have an impaired gut microbiome. These are likely to impact on both physical and mental health. For example, reduced physical activity in patients with cardiovascular diseases may exacerbate depressive symptoms [4], while anxiety-related eating disorders (eg, binge eating) may further worsen metabolic dysfunction.
4. Side effects and drug-drug interactions of medications can occur during treatment. Some medications for chronic physical illnesses may have side effects that impact normal brain function and behavior (eg, appetite). Similarly, some medications for mental disorders may increase the risk of developing certain chronic physical illnesses. As people develop more comorbid conditions, their range of prescribed drugs increase, thus increasing the risk of even more comorbidity; for example, antipsychotic medications (eg, olanzapine) may indirectly increase the risk of diabetes through metabolic syndrome [3].

Thus, the mechanism of comorbidity between chronic physical illnesses and mental disorders is highly complex and more research is warranted to understand and identify the underlying associations. This will only be successful through research approaches that are holistic and consider biological, genetic, sociological, psychological, and environmental aspects that could interact to drive the loss of health and the pathways to multimorbidity.

The relationship between well-being and emotional status and physical health, and vice versa, is an important source of inspiration for the study of comorbid mechanisms of chronic physical illnesses and mental health disorders. In traditional Chinese medicine (TCM), emotional status is closely related to human health. For example, TCM emphasizes the impact of “emotional injuries” on organ function, such as “anger injuring the liver” or “worry injuring the spleen.” In this study, the high comorbidity between “gastrointestinal diseases and neurotic stress disorders” (RR=1.821) aligns with the TCM theory of “liver qi stagnation and spleen deficiency,” where prolonged anxiety (liver qi stagnation) leads to digestive dysfunction (spleen deficiency). Furthermore, TCM’s holistic approach supports the bidirectional interaction between chronic physical and emotional conditions. For instance, in patients with diabetes, the “dual deficiency of qi and yin” may exacerbate depressive symptoms. An adverse emotional state can negatively affect physical health and function, leading to the development of comorbidity of both chronic physical illnesses and mental health disorders. The rigorous compartmentalization of Western medicine has led to great progress being made through specialization. However, it is perhaps only now fully realizing that taking a more holistic approach that considers the integration and interaction of all biological, behavioral, and environmental components is a sensible approach to take in parallel. This has already started to reveal great benefits in systems biology, where the interactions between genes, proteins, metabolites, and external/environmental factors hold the key to a full appreciation of how health and illness relate to each other.

### Age and Gender Differences in Comorbidities

Comorbidity of chronic physical illnesses with mental health disorders affects people of all ages, including hospitalized children and adolescents (age ≤18 years). Consistent with previous studies, we found that the association between chronic physical illness and mental health disorders varied across age-sex patient groups [16,31,32]. In our study, although the same combinations of comorbidities were present in different age-sex patient groups (eg, esophagus, stomach, and duodenum diseases with neurotic stress–related and somatoform diseases), the differences between the individual patient groups were notable.

Several combinations of disorders exhibited high comorbidity intensities specific to certain patient populations, such as dyslipidemia and schizophrenia with delusional diseases; esophagus, stomach, and duodenum diseases with organic mental disorders; prostate diseases with neurotic stress–related and somatoform disorders; and heart failure with neurotic stress–related and somatoform disorders. Our findings emphasize the importance of attention to the co-prevention of specific mental disorders with chronic physical illnesses in specific age groups, particularly in the male population aged 45‐60 years. For example, for male patients with cardiovascular disease aged 45‐60 years, it is recommended to conduct annual Patient Health Questionnaire-9 depression screenings in conjunction with the Framingham risk score to assess comorbidity risk. Alternatively, community hospitals could promote “dual heart medicine” outpatient clinics, which simultaneously manage both cardiovascular disease and anxiety/depression.

### Strengths and Limitations

The primary strengths of this study can be summarized as follows. First, this study provides an inaugural regional investigation that evaluates comorbidity between chronic physical illnesses and mental health disorders by analyzing patient longitudinal electronic medical records. The study pinpoints a number of chronic physical illnesses with an elevated risk of association with mental health disorders and scrutinizes patterns of comorbidity, including variations by age and gender. Furthermore, this method places a strong emphasis on considering temporal dependencies of disease onset by using ML classification models. This allows us to gain a comprehensive understanding of the relationships between various chronic physical illnesses and mental health disorders in patients before they are diagnosed with a mental illness. Importantly, our approach is not limited to specific studies, but can be extended to other health care datasets to analyze comorbidity patterns.

By applying this method to diverse datasets, we can broaden our understanding of the complex relationships between diseases. This provides a broader and deeper insight into medical research and clinical practice.

This study has several limitations. First, a primary limitation is the unavailability of individual-level socioeconomic status, lifestyle factors, clinical data, and treatment information, all of which are crucial in fully comprehending distinctions between comorbidities. Second, since the recording of diseases in clinical data may lead to incomplete diagnoses, this study used “real-world” medical care data. The quality of disease coding lies beyond our control, and variations in recording among physicians and at different time points may result in missing or inaccurate information. Third, we only included diseases with a prevalence of ≥1% within this group, and the patients examined were primarily from a regional group in China, constituting a singular data source. The specific comorbidities and risk analyses necessitate further clinical validation.

To address the discussed limitations, our future research will collaborate with community health service centers to obtain data on patients’ education levels, income, and insurance types, and construct a multisource database (eg, electronic health records combined with social service registrations). Additionally, to address the varying quality of diagnostic codes, we plan to improve data reliability through multicenter clinical audits (eg, random sampling of 10% of cases for review by specialist physicians).

### Comparison With Prior Work

This study builds on existing research while providing new insights into the comorbidity between chronic physical illnesses and mental health disorders. In comparison to previous studies, the key similarities and differences are as follows.

Our study findings show a high degree of consistency with those from Western population studies, for example, the comorbidity patterns observed between cardiovascular diseases (such as hypertension and heart failure) and mental health disorders are consistent with those reported in the UK cohort by Launders et al [1]. This alignment enhances the generalizability of our findings across different populations. At the same time, our findings resonate with previous research in China. The dietary habits of the Chinese population (eg, high salt intake) and sociopsychological stress may exacerbate the interaction between metabolic diseases (eg, diabetes) and mental health disorders, as noted by Chen et al [22]. This also supports the 4 potential mechanisms we proposed.

Additionally, we identified some unique findings, such as a strong association between prostate disease and organic mental disorders (RR=2.055), which is less commonly observed in Western studies. This finding may reflect the distinct disease burden in China’s aging male population, offering a new perspective on comorbidity issues in this demographic.

### Conclusions

In this paper, we presented a regional study in Southern China focusing on the comorbidity and risk association between chronic physical illnesses and mental health disorders. We modeled patients’ disease trajectories and analyzed the risk of comorbidity between mental health disorders and chronic physical illnesses. Through ML-based predictive modeling, we evaluated chronic physical illnesses that exhibited a higher risk of comorbidity with mental disorders. Additionally, we analyzed the risk of comorbidities, considering age and gender differences in comorbidity patterns. The findings revealed that in the male patient population aged 45‐60 years, there was a stronger interaction and higher risk of comorbidities between chronic physical illnesses and mental health disorders. Our research findings support the need for implementing “tailored” disease prevention and management measures based on patient age and gender in clinical prevention and care management. Our study particularly highlights the significance of age and gender in comorbidity research between chronic physical illnesses and mental disorders in a Chinese population.

## Abbreviations

AUC: area under the curve
CVD: cerebrovascular diseases
DL: dyslipidemia
DM: diabetes
DMD: depression and mood disorders
ESD: esophagus, stomach, and duodenum diseases
ExtRaTrees: extremely randomized trees
GERD: gastroesophageal reflux disease
HF: heart failure
HTN: hypertension
ICD-10: *International Classification of Diseases, Tenth Revision*
IHD: ischemic heart disease
Light GBM: light gradient boosting machine
MR: mental developmental disorders
NSRSD: neurotic stress-related and somatoform diseases
OMD: other metabolic diseases
OMD_F: organic mental disorders
PD: prostate diseases
PDD: pervasive developmental disorders
RF: random forest
ROC: receiver operating characteristic
RR: relative risk
SD: sleep disorders_F
SDD: schizophrenia and delusional disorders
SUD: Mental and Behavioral Disorders Due to Psychoactive Substance Use
TCM: traditional Chinese medicine
XGBoost: extreme gradient boosting
